# Inflammatory targeted nanoplatform incorporated with antioxidative nano iron oxide to attenuate ulcerative colitis progression

**DOI:** 10.1016/j.isci.2025.112448

**Published:** 2025-04-16

**Authors:** Haojun Chen, Wei Sun, Can Li, Qiuyang Wang, Xucai Wang, Yingjie Du, Wenbo Chen, Min Wang, Caoxing Huang, Rong Wang

**Affiliations:** 1 Co-Innovation Center for Efficient Processing and Utilization of Forest Resources, College of Chemical Engineering, Nanjing Forestry University, Nanjing 210037, China; 2 Department of Orthopedics, The Jiangyin Clinical College of Xuzhou Medical University, 163 Shoushan Road, Jiangyin 214400, Jiangsu, P.R. China; 3 School of Artificial Intelligence and Information Technology, Nanjing University of Chinese Medicine, Nanjing 210023, China; 4 State Key Laboratory of Pharmaceutical Biotechnology, Department of Sports Medicine and Adult Reconstructive Surgery, Nanjing Drum Tower Hospital, The Affiliated Hospital of Nanjing University Medical School, Nanjing 210008, China; 5 Department of Wood Science, The University of British Columbia, 2424 Main Mall, Vancouver, BC V6T 1Z4, Canada; 6 Digestive Endoscopy Department, The First Affiliated Hospital with Nanjing Medical University & Jiangsu Province Hospital, Nanjing 210000, China; 7 Jiangsu Province Key Laboratory for Molecular and Medical Biotechnology, College of Life Science, Nanjing Normal University, Nanjing 210046, Jiangsu, China

**Keywords:** Health sciences, Materials science, Biomedical materials

## Abstract

Antioxidative nanomaterials with reactive oxygen species (ROS) scavenging capabilities hold promise for the treatment of ulcerative colitis (UC). However, their clinical application is limited by rapid diffusion, susceptibility to inactivation, and insufficient targeting of inflammatory sites. This study focuses on developing a nanoplatform by integrating iron oxide nanoparticles (IONPs) into zeolitic imidazolate frameworks-8 (ZIF-8), termed as ZIF-8@IONPs. ZIF-8@IONPs exhibited good biocompatibility and effective ROS scavenging capabilities in RAW 264.7 cells. To enhance inflammatory targeting, HA@ZIF-8@IONPs were generated through hyaluronic acid (HA) surface modification. HA@ZIF-8@IONPs effectively reduced damage to intestinal tissues in the UC mouse model. Mechanistic revealed that HA@ZIF-8@IONPs exhibited antioxidant and anti-inflammatory activities by eliminating endogenous ROS, activating the Nrf2 signaling pathway, and inhibiting the NF-κB signaling pathway. This study highlights the nanoplatform’s potential as a promising candidate for UC treatment due to its great targeting of inflammatory microenvironments and efficient ROS scavenging.

## Highlights

• ZIF-8@IONPs were prepared to scavenge ROS and enhance biocompatibility for UC
• HA@ZIF-8@IONPs selectively targeted inflamed intestines and reduced damage in UC mice
• HA@ZIF-8@IONPs mitigated UC via ROS modulation, Nrf2 activation, and NF-κB inhibition

## Graphical abstract

**Figure:**
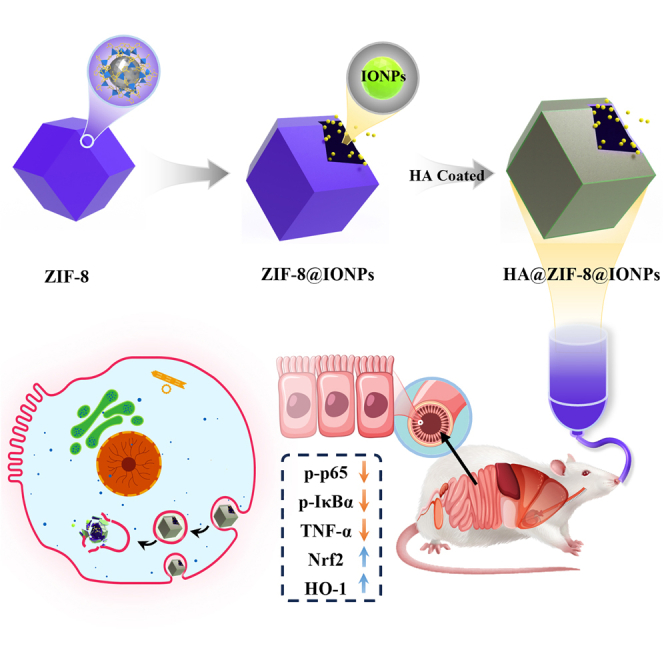

## Introduction

Ulcerative colitis (UC) is a chronic inflammatory bowel disease of unknown etiology.^1^ Its primary clinical symptoms include abdominal pain, tenesmus, and bloody diarrhea, often characterized by recurrent flare-ups.^2^ The global incidence and prevalence of UC have been steadily rising, posing a substantial global health burden due to its increased risk of cancer and mortality compared to other colonic inflammatory disorders.^3^ Although the precise pathogenesis of UC remains unclear, it is believed to involve multiple factors, including intestinal microbiota dysbiosis, abnormal immune responses, dysfunction of the intestinal mucosal barrier, infections, and genetic predispositions.^4^ Standard treatment primarily aims to achieve and sustain corticosteroid-free remission. Oral and rectal 5-aminosalicylates are frequently used for treating mild to moderate UC. In more severe cases, treatment options include biologics targeting tumor necrosis factor and integrins, thiopurines, and small-molecule Janus kinase inhibitors.^5^ However, conventional oral drug delivery systems encounter significant challenges in the gastrointestinal tract, including exposure to harsh environmental conditions, such as highly acidic gastric juice, digestive enzymes, and varying bacterial populations, which can compromise drug stability and reduce therapeutic efficacy.^6^ Therefore, the development of safe and effective treatment strategies for UC remains an urgent priority in medical research, holding both clinical and scientific significance.

Emerging evidence indicates that reactive oxygen species (ROS) play a pivotal role in the development and progression of UC.^7^ Persistent intestinal inflammation in UC patients leads to excessive ROS production, causing damage to DNA, proteins, and lipids within intestinal epithelial cells. This cellular damage induces apoptosis or necrosis, further compromising the integrity of the intestinal mucosal barrier.^8^ Excessive ROS can stimulate immune cells, including macrophages and neutrophils, to release pro-inflammatory cytokines, such as tumor necrosis factor-α (TNF-α), IL-6, and IL-1β, thereby exacerbating localized inflammation and contributing to UC pathogenesis.^9^ Hence, controlling oxidative stress and reducing ROS production may represent important therapeutic strategies for alleviating UC.

Recently, functional nanomaterials have introduced significant innovations in biomedical applications due to their biocompatibility and unique physicochemical properties, such as rich surface chemistry and catalytic activities.^10^^,^^11^^,^^12^ Various nanomaterials, including iron oxide nanoparticles (IONPs), cerium oxide nanoparticles (CeNPs), and ultrasmall Prussian blue nanoparticles (PbNPs), have demonstrated the ability to remove ROS and reduce oxidative stress within inflammatory microenvironments.^13^^,^^14^^,^^15^ IONPs, the only inorganic functional nanoparticles approved by the FDA, are extensively utilized in the treatment of various inflammatory diseases due to their ROS scavenging properties and superior biocompatibility.^16^ Our previous research showed that polyglucose-sorbitol-carboxymethyl ether (PSC)-modified IONPs enhanced antioxidative activity in MC3T3-E1 and RAW 264.7 cells, facilitating ROS scavenging. Moreover, they promoted osteogenic differentiation via Akt-GSK-3β-β-catenin activation and inhibited osteoclast differentiation by suppressing the MAPK and NF-κB pathways *in vitro*.^17^ However, several challenges persist in using IONPs alone for UC treatment, including rapid diffusion, short duration of therapeutic effect, susceptibility to inactivation within the gastrointestinal tract, and suboptimal targeting to the affected sites.

IONPs can be incorporated into biocompatible delivery systems, such as hydrogels, metal-organic frameworks (MOFs), or polymer-based nanoparticles, to overcome these challenges. This approach may improve retention at the target site, enhance drug stability in the gastrointestinal tract, and enable controlled, sustained release, thus boosting therapeutic efficacy in UC treatment.^18^^,^^19^ MOFs, synthesized through coordination reactions between metal cluster ions and organic ligands, are recognized as effective drug delivery systems in biomedical applications. Their tunable porosity, high surface area, and versatile functionality enhance the stability and controlled release of therapeutic agents.^20^^,^^21^^,^^22^ Zeolitic imidazolate frameworks-8 (ZIF-8), composed of Zn^2+^ and 2-methylimidazole (2-MA), is among the most widely used MOFs due to its unique structural features and exceptional properties. The superior structural properties and controlled collapse characteristic of ZIF-8 make it an ideal carrier for transporting various guest substances, such as functional nanoparticles, anti-tumor and antibacterial drugs, proteins, and nucleic acids.^23^ One advantage of ZIF-8 is its capacity for surface modification with different functional molecules. Hyaluronic acid (HA) targets the CD44 receptor, which is present at low levels on epithelial, hematopoietic, and neuronal cells but is significantly upregulated in inflamed areas. This selective targeting capability makes HA a promising candidate for delivering therapeutic agents to inflamed tissues.^24^ Consequently, HA modification on ZIF-8-loaded IONPs enhances targeting to inflammatory microenvironments, suggesting significant potential for UC therapy.

**Scheme 1:**
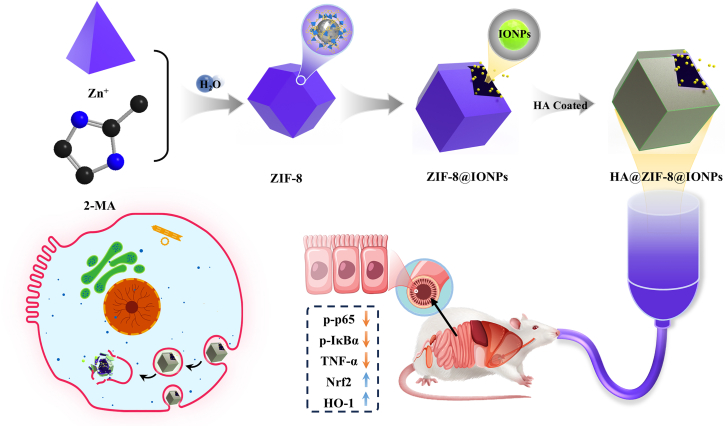
Schematic illustration of the HA-modified antioxidative nano-iron oxide incorporated nanoplatform with excellent inflammatory microenvironment-targeting ability to attenuate the progression of ulcerative colitis

In this work, antioxidant IONPs were incorporated into ZIF-8 to form a smart nanoplatform, referred to as ZIF-8@IONPs. The morphological and physicochemical properties, *in vitro* biocompatibility, and ROS scavenging ability of the nanoplatform were comprehensively assessed. Furthermore, surface modification with HA further enhanced the targeting ability of ZIF-8@IONPs toward the inflammatory microenvironment (HA@ZIF-8@IONPs), making it a promising system for precise therapeutic delivery. The therapeutic efficacy of HA@ZIF-8@IONPs was further evaluated using a mouse model of UC *in vivo*. This study demonstrated that this novel nanoplatform, with excellent inflammatory microenvironment-targeting and ROS scavenging ability, can be considered a potential candidate for UC treatment (Scheme 1).

## Results and discussion

### Synthesis and characterization of ZIF-8@IONPs

**Figure 1:**
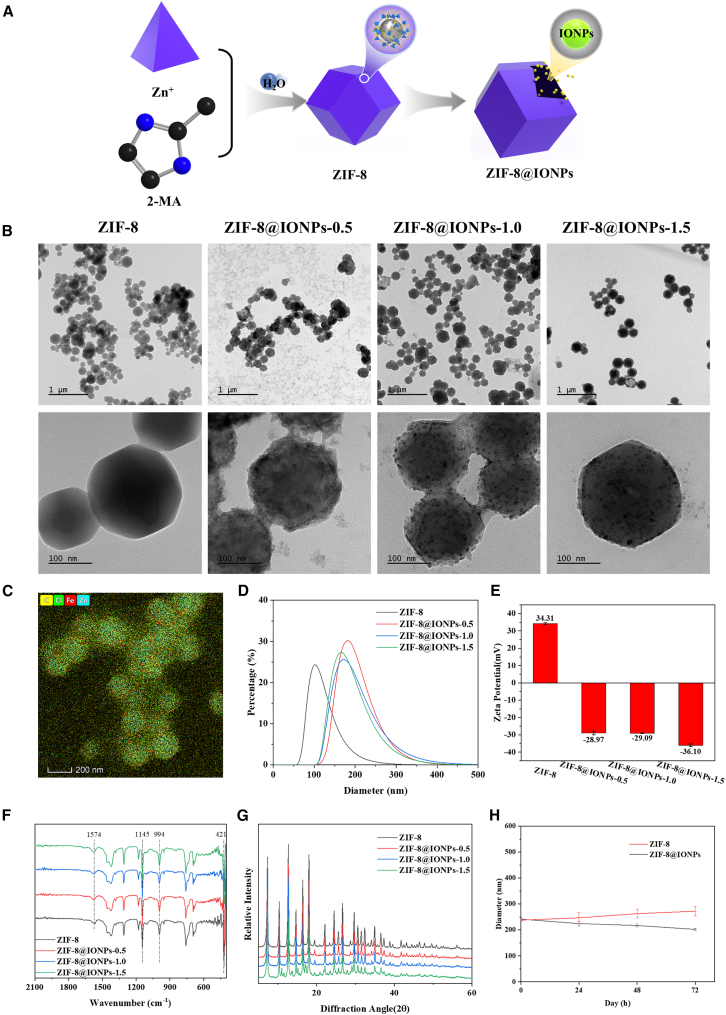
Synthesis and characterization of ZIF-8@IONPs (A) Schematic representation of the synthesis of ZIF-8@IONPs. (B) TEM images of pure ZIF-8 and ZIF-8@IONPs. (C) Elemental mapping of ZIF-8@IONPs. (D) Dynamic light scattering (DLS) analysis of the hydrated diameter and distribution of ZIF-8@IONPs nanoparticles. (E) Zeta potential of ZIF-8@IONPs nanoparticles (*n* = 3 per group). (F) FT-IR spectra of ZIF-8@IONPs. (G) XRD pattern of ZIF-8@IONPs. (H) The hydrated diameters of ZIF-8 and ZIF-8@IONPs nanoparticles at different time periods analyzed by DLS (*n* = 3 per group).

ZIF-8 was employed to encapsulate IONPs within its hollow structure to reduce the rapid diffusion and limited therapeutic duration of IONPs in the treatment of UC. As shown in Figure 1A, the ZIF-8@IONPs composite was synthesized via a one-pot method, successfully incorporating IONPs into the ZIF-8 framework. The study investigated the influence of varying IONP concentrations (0.5 mg/mL, 1.0 mg/mL, and 1.5 mg/mL) on the morphological and physicochemical properties of ZIF-8@IONPs. Figure 1B presents transmission electron microscopy (TEM) images of both ZIF-8 and ZIF-8@IONPs. The particle size of pure ZIF-8 was approximately 100 nm, while the ZIF-8@IONPs composites, at all three IONP concentrations, exhibited sizes ranging from 150 to 200 nm—slightly larger than the ZIF-8 control group. Despite this size difference, the morphology of the IONP-loaded groups remained largely unchanged compared to the ZIF-8 group. To further verify the successful incorporation of IONPs, energy-dispersive spectroscopy (EDS) elemental mapping is employed and the results demonstrate that iron (Fe) has been incorporated in the nanoparticles (Figures 1C and S1). Since the precursor of ZIF-8 (2-methylimidazole and zinc nitrate) don’t contains iron, the Fe signals detected in ZIF-8@IONPs exclusively originate from the IONPs, which indicated that the IONPs have been successfully incorporated into the ZIF-8 structure. The size distributions, evaluated from the TEM images, are shown in Figure S2. The particle sizes of ZIF-8 loaded with different IONP concentrations were slightly larger than those of the blank group, but the effect of varying IONP concentrations on particle size was not significant. Dynamic light scattering (DLS) measurements (Figure 1D) revealed a slight increase in the hydrodynamic diameter of ZIF-8@IONPs compared to blank ZIF-8, although the effect of IONP concentration on particle size was minimal, with sizes consistently ranging from 150 to 200 nm across all IONP concentrations. Zeta potential analysis (Figure 1E) indicated that ZIF-8, which is positively charged (34.31 mV), became negatively charged upon binding with IONPs. Furthermore, the absolute value of the zeta potential increased with higher IONP concentrations, suggesting a stronger negative charge as more IONPs were incorporated. Fourier transform infrared (FT-IR) spectroscopy (Figure 1F) exhibited the characteristic stretching vibrations of the imidazole ring (C=N at 1574 cm^−1^, C–N at 1145 cm^−1^, and 994 cm^−1^) in both ZIF-8 and ZIF-8@IONPs. Additionally, the Zn-N stretching mode of ZIF-8 appeared at 421 cm^−1^. The X-ray diffraction (XRD) pattern (Figure 1G) demonstrated that the diffraction peaks of ZIF-8@IONPs corresponded with those of ZIF-8, confirming the retention of the ZIF-8 crystal structure in ZIF-8@IONPs. The crystal structure of ZIF-8 remained intact following the incorporation of IONPs, as evidenced by the preservation of the characteristic diffraction peaks. These structural characterization results suggest that varying concentrations of IONPs did not alter the chemical bonding nor significantly impact the crystal structure of ZIF-8. To further test the *in vitro* stability of ZIF-8 and ZIF-8@IONPs, their hydrodynamic diameter was measured at 0, 24, 48, and 72 h. As shown in Figure 1H, the results indicated that there was no significant changes in the hydrodynamic diameter of ZIF-8 and ZIF-8@IONPs across these time points, demonstrating the excellent stability of all prepared nanoparticle.

### *In vitro* and *in vivo* biocompatibility, ROS scavenging, and anti-inflammatory activities of ZIF-8@IONPs

**Figure 2:**
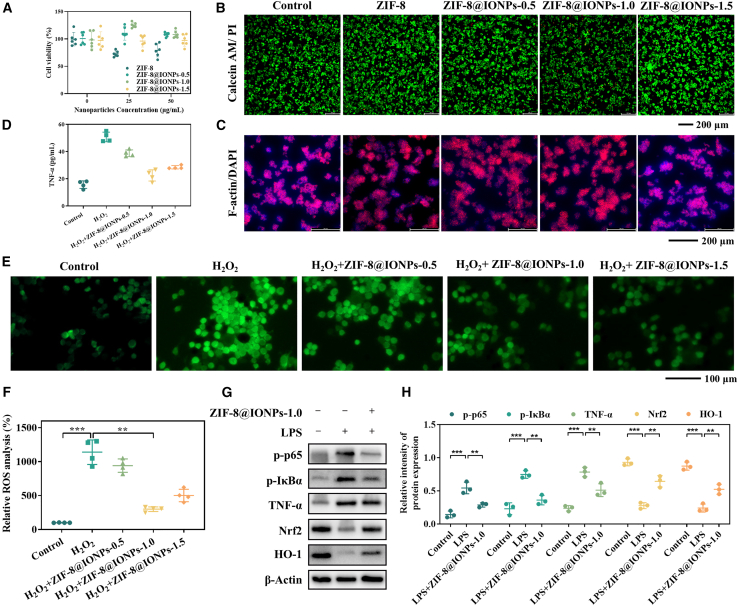
Biocompatibility, ROS scavenging ability, and anti-inflammatory activities of ZIF-8@IONPs (A) Cytotoxicity of ZIF-8@IONPs on RAW 264.7 cells after a 24-h co-incubation, as assessed by the cell counting Kit-8 (*n* = 6 per group). (B) Cytotoxicity of ZIF-8@IONPs after a 24-h co-incubation in RAW 264.7 cells, examined using the calcein-AM/PI staining kit (scale bar, 200 μm). (C) Cytoskeletal analysis of RAW 264.7 cells after 24-h co-incubation, stained with phalloidin (scale bar, 200 μm). (D) Measurement of secretion of TNF-α by cells by ELISA assay. (E) ROS fluorescence staining to evaluate the ROS-scavenging ability of ZIF-8@IONPs nanoparticles in RAW 264.7 cells. (F) Semi-quantitative analysis of immunofluorescence staining for ROS (*n* = 4 per group). (G) Western blot analysis showing the effects of ZIF-8@IONPs-1.0 on p-p65, *p*-IκBα, TNF-α, Nrf2, and HO-1 signaling pathways, with β-actin as an internal reference. (H) Levels of colitis-related inflammatory cytokines, including p-p65, *p*-IκBα, TNF-α, Nrf2, and HO-1, in various groups (*n* = 3 per group).

The objective of this study was to develop a biocompatible smart nanoplatform with anti-inflammatory properties for the treatment of UC. To assess its biosafety, the cytotoxicity of ZIF-8@IONPs was first evaluated in RAW 264.7 cells. According to the results of the CCK-8 assay (Figure 2A), pure ZIF-8 exhibited minimal cytotoxicity following a 24-h co-incubation with RAW 264.7 cells. ZIF-8@IONPs demonstrated negligible cytotoxicity at a concentration of 50 μg/mL, likely due to the incorporation of biocompatible IONPs. Live/dead staining with fluorescent dyes was conducted to visualize the cytotoxic effects of ZIF-8@IONPs on RAW 264.7 cells, differentiating viable from non-viable cells. This technique allows for clear visualization of viable cells in green and non-viable cells in red, providing a direct assessment of cell survival following treatment.^25^ As shown in Figures 2B and S3, the majority of cells exhibited green fluorescence, indicating cell viability following treatment with ZIF-8@IONPs. Furthermore, F-actin/DAPI staining was employed to examine the effects of ZIF-8@IONPs on the cytoskeleton and nuclear morphology of the cells. The red fluorescence (F-actin) highlighted filamentous actin, showing well-defined cell boundaries and cytoskeletal organization, while the blue fluorescence (DAPI) stained the nuclei, confirming the presence of intact and healthy cells.^26^ As depicted in Figures 2C and S4, RAW 264.7 cells treated with ZIF-8@IONPs displayed a uniform distribution of F-actin throughout the cytoplasm, with well-rounded, distinct nuclei, indicating normal cell morphology, and structural integrity.

To further assess the impact of ZIF-8@IONPs on inflammatory responses of RAW 264.7 cells, TNF-α was measured through enzyme-linked immunosorbent assay (ELISA). As shown in Figure 2D, there was a significant increase in the levels of TNF-α after the H_2_O_2_ treatment. Among these nanoparticles, the ZIF-8@IONPs-1.0 group exhibited the lowest TNF-α secretion level compared to other treatment groups, demonstrating it possessed the superior anti-inflammatory efficacy. Excessive ROS production plays a central role in the pathogenesis and progression of UC, and therapies targeting ROS scavenging can mitigate oxidative stress, protect the intestinal barrier, and disrupt the inflammation cycle in UC.^27^ To evaluate the ROS-scavenging ability of ZIF-8@IONPs, cells were exposed to hydrogen peroxide to induce ROS production, and fluorescence intensities were compared to reflect intracellular ROS levels across different groups. Higher green fluorescence intensity corresponds to increased intracellular ROS levels. As shown in Figures 2E and 2F, the fluorescence intensities of the ZIF-8@IONPs-1.0 and ZIF-8@IONPs-1.5 groups were significantly lower than those of the other groups, indicating enhanced ROS scavenging capabilities in these formulations. We further assessed the expression of key proteins involved in inflammatory signaling pathways to investigate the anti-inflammatory effects of ZIF-8@IONPs at the cellular level. An inflammatory response was induced in the cells using lipopolysaccharide. As shown in Figures 2G and 2H, there was a significant increase in the levels of p-p65, *p*-IκBα, and TNF-α, while the expression of Nrf2 and HO-1 was markedly reduced compared to the normal controls. Treatment with ZIF-8@IONPs-1.0 inhibited the activation of the NF-κB pathway and concurrently enhanced the expression of detoxification enzymes and antioxidant proteins, including Nrf2 and HO-1, which are regulated by this pathway. These results suggest that ZIF-8@IONPs not only reduce oxidative stress but also improve inflammatory responses at the molecular level. Furthermore, the *in vivo* biocompatibility of ZIF-8@IONPs was further investigated. As shown in Figure S5, the serum biochemical parameters of alanine aminotransferase (ALT), aspartate aminotransferase (AST), and albumin (ALB) were remained within the established murine reference ranges in both the control and experimental treatment groups. These results indicated that the prepared ZIF-8@IONPs possessed good biocompatibility. Histopathological analysis of hematoxylin and eosin (H&E)-stained tissue sections (Figure S6) also confirmed these issues. Specifically, myocardial sections exhibited spindle-shaped myocardial fibers with well-defined striations. Hepatic sections displayed hepatocytes arranged radially around the central veins within hepatic lobules. Splenic sections demonstrated lymphocyte aggregates forming distinct blue-stained nodules. Pulmonary sections showed occasional blue-stained neutrophils within alveolar spaces. Renal sections revealed uniformly distributed glomeruli. Importantly, no statistically significant differences were observed between the control and treatment groups. Overall, both the analysis results from serum biochemical parameters and H&E-stained tissue sections provided the strong evidence supporting the excellent *in vivo* biocompatibility of ZIF-8@IONPs.

### Surface modification of HA to target inflammation

**Figure 3:**
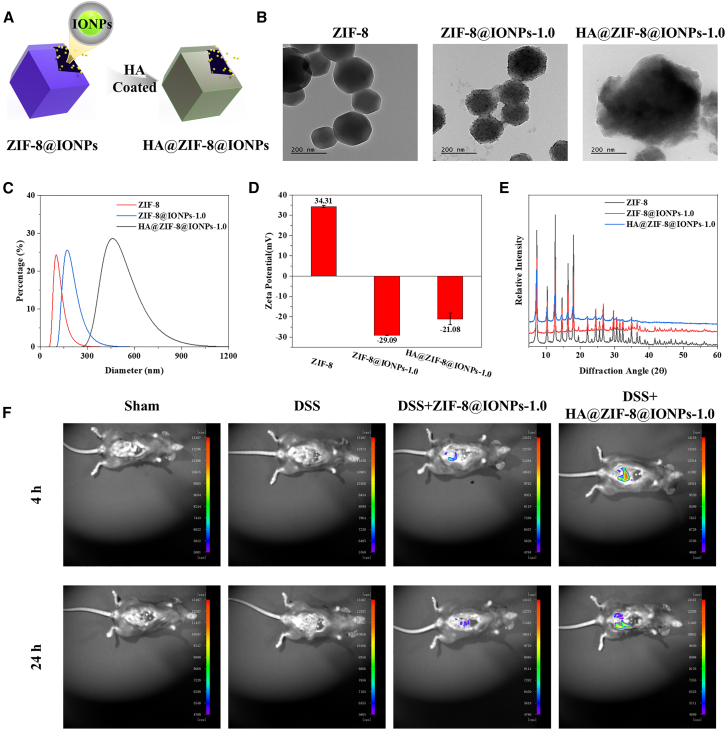
Surface modification of HA to target inflammation (A) Synthesis of HA@ZIF-8@IONPs. (B) TEM image of HA@ZIF-8@IONPs. (C) DLS measurements of the hydrated diameter and distribution of HA@ZIF-8@IONPs. (D) Zeta potential of HA@ZIF-8@IONPs (*n* = 3 per group). (E) XRD pattern of HA@ZIF-8@IONPs. (F) Fluorescence distribution in mice following the administration of rhodamine B-labeled ZIF-8@IONPs and HA@ZIF-8@IONPs at different time points.

Targeting the inflammatory microenvironment is critical for the efficacy of ROS-scavenging nanomaterials in inflammatory-related diseases, as it ensures that these nanomaterials accumulate at sites of high oxidative stress, thereby enhancing their therapeutic efficacy while minimizing off-target effects.^28^^,^^29^ Recent studies have increasingly demonstrated that HA interacts with several receptors on the surface of immune cells, particularly CD44 and RHAMM, which are often upregulated in inflamed tissues. This upregulation allows HA to preferentially bind to these receptors, facilitating targeted delivery to sites of inflammation.^30^ To enhance the targeting capability of ZIF-8@IONPs for the inflammatory microenvironment, surface modification with HA was employed, as schematically depicted in Figure 3A. TEM images (Figure 3B) of ZIF-8, ZIF-8@IONPs, and HA@ZIF-8@IONPs revealed that the HA coating significantly enlarges the ZIF-8@IONPs nanoparticles. This observation is further supported by the hydrated particle size data presented in Figure 3C. HA coats the outer surface of ZIF-8@IONPs, and due to its negative charge, the zeta potential of HA@ZIF-8@IONPs, shown in Figure 3D, remains negative. However, the negative charge of HA is weaker than that of IONPs, resulting in a lower absolute zeta potential for HA@ZIF-8@IONPs compared to ZIF-8@IONPs. As indicated by the X-ray diffraction (XRD) pattern in Figure 3E, the characteristic peak of ZIF-8 remains, although its intensity is significantly reduced. The observed attenuation in characteristic peak intensity, combined with TEM morphological analyses, enlarged hydrated particle dimensions, and modified surface charge characteristics, collectively demonstrate successful HA encapsulation on ZIF-8@IONPs surfaces, thereby validating the nanocomposite synthesis achieved through this methodology.

To further assess the targeting capability and retention duration of HA@ZIF-8@IONPs in the inflammatory microenvironment, bioimaging technology was employed to track the localization and retention time of HA@ZIF-8@IONPs after conjugation with rhodamine B. As shown in Figures 3F and S7, no fluorescence was observed in the colons of both normal mice and those in the dextran sulfate sodium(DSS)-induced UC model group. At the 4-h time point following the final administration, fluorescence was detected in the colons of both the ZIF-8@IONPs and HA@ZIF-8@IONPs groups. Notably, the HA@ZIF-8@IONPs group exhibited significantly higher fluorescence intensity compared to the ZIF-8@IONPs group. After 24 h, the HA@ZIF-8@IONPs group maintained prominent fluorescence in the colon, whereas the fluorescence intensity in the ZIF-8@IONPs group had significantly decreased and was notably lower than that of the HA@ZIF-8@IONPs group. These results confirm that the HA coating enhances the ability of ZIF-8@IONPs to target colonic inflammatory lesions and prolongs their retention time within these lesions.

### HA@ZIF-8@IONPs alleviate colitis and relieve histopathological symptoms in the DSS-induced UC mouse model

**Figure 4:**
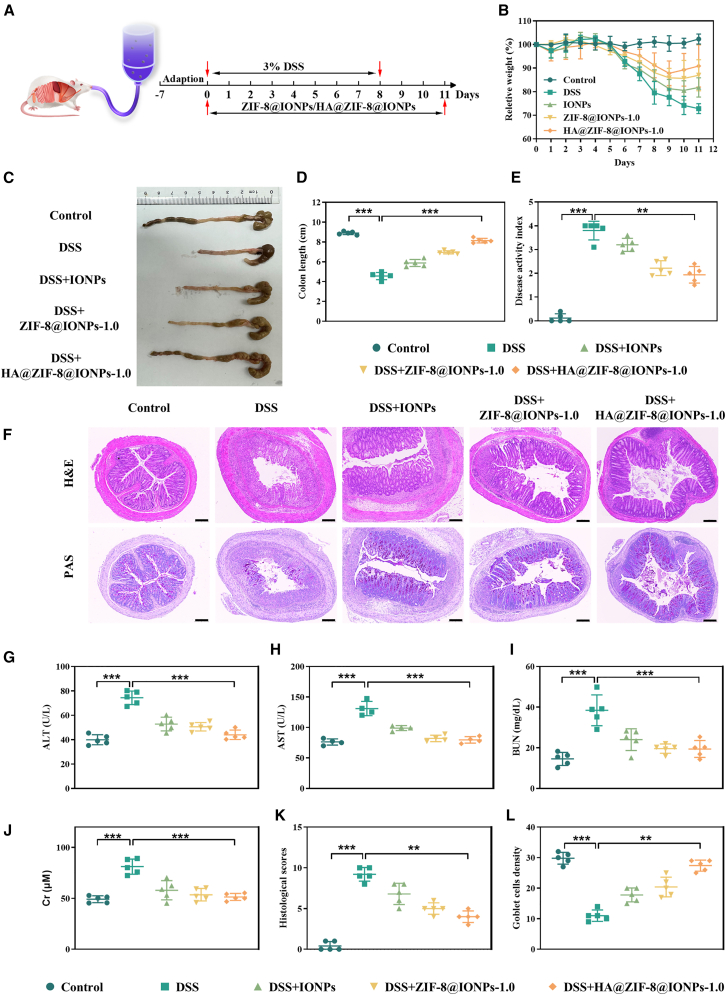
HA@ZIF-8@IONPs alleviate colitis in DSS-fed mice (A) Mice were intragastrically administered the control vehicle, ZIF-8@IONPs, or HA@ZIF-8@IONPs at a daily dose of 100 mg/kg body weight for 14 consecutive days. (B–E) (B) Body weight, (C) macroscopic phenotype, (D) colon length, and (E) disease activity index of mice (*n* = 5 per group). (F) Representative sections following H&E staining and PAS staining (scale bar, 200 μm). (G–L) (G) AST, (H) ALT, (I) BUN, (J) Cr, (K) histology scores, and (L) goblet cell density in the serum (*n* = 5 per group). Asterisk indicates statistically significant differences between control and experience group (∗∗p < 0.01; ∗∗∗p < 0.005).

To investigate the potential therapeutic efficacy of IONPs, ZIF-8@IONPs, and HA@ZIF-8@IONPs *in vivo* against DSS-induced acute colitis, we established a murine model using 3% DSS exposure (Figure 4A), a well-established method for studying acute UC. After treatment with IONPs, ZIF-8@IONPs, or HA@ZIF-8@IONPs, a significant increase in body weight was observed in DSS-induced colitis mice (Figure 4B). Furthermore, these groups exhibited a moderate extension in colon length (Figures 4C and 4D). Mice with DSS-induced UC displayed notable clinical symptoms, including weight loss, lethargy, diarrhea, unkempt fur, and fecal occult blood, resulting in higher DAI scores compared to control mice. Pretreatment with IONPs, ZIF-8@IONPs, or HA@ZIF-8@IONPs significantly alleviated these symptoms and reduced DAI scores (Figure 4E). Importantly, all treatment groups demonstrated pharmacological efficacy in managing DSS-induced UC, with HA@ZIF-8@IONPs showing the most pronounced effect.

Compared to normal controls, the UC model group exhibited distinct inflammatory histopathological features, such as severe colonic mucosal damage, substantial epithelial erosion, depletion of goblet cells, crypt distortion or loss, and extensive infiltration of inflammatory cells (Figure 4F). Consequently, this group displayed elevated histological scores and a reduced density of goblet cells (Figures 4K and 4L). Notably, treatment with IONPs, ZIF-8@IONPs, or HA@ZIF-8@IONPs ameliorated the severe histopathological damage induced by DSS. This was evident by well-preserved crypt structures, a higher density of goblet cells, intact gut mucosa, and significantly lower histological scores compared to the DSS-induced colitis group. The enhanced bioactivity and targeting capabilities of HA@ZIF-8@IONPs, likely attributed to their smaller particle size, contribute to their superior efficacy in alleviating UC. Furthermore, serum biochemical markers for liver and kidney function, including ALT (Figure 4G), AST (Figure 4H), blood urea nitrogen (BUN) (Figure 4I), and creatinine (Cr) (Figure 4J), were examined, and no signs of impairment were observed. These results suggest that these treatments exhibit favorable safety profiles.

### HA@ZIF-8@IONPs reduced inflammation and oxidative stress

**Figure 5:**
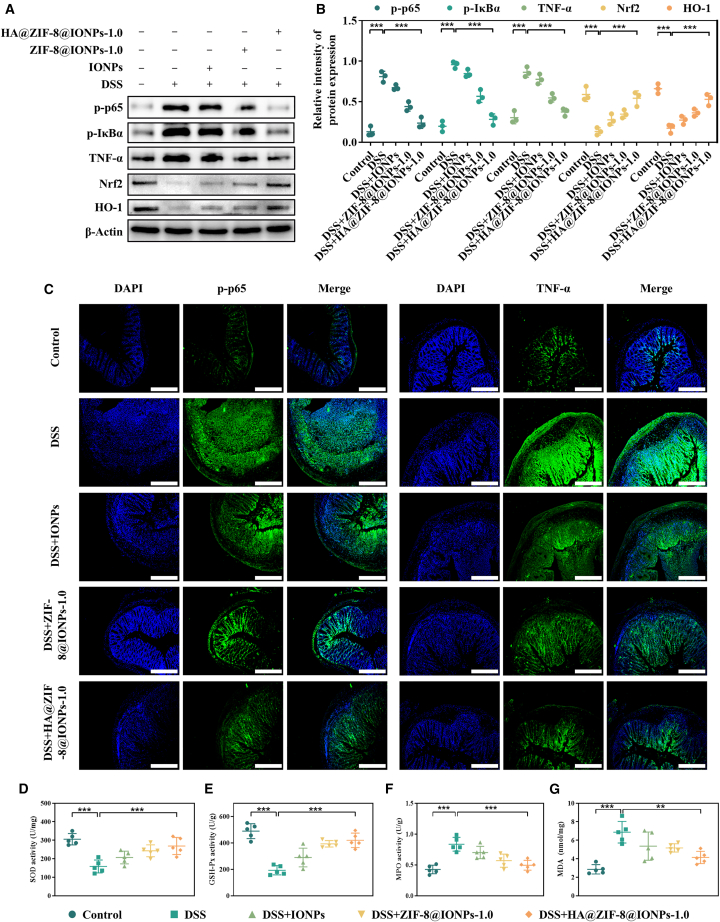
HA@ZIF-8@IONPs exhibited anti-inflammatory and antioxidant activities via activation of the Nrf2 and inhibition of the NF-κB pathways (A) The expression levels of p-p65, *p*-IκBα, TNF-α, Nrf2, and HO-1 in colonic tissues were assessed using western blots, with β-Actin as an internal reference. (B) the relative intensities of p-p65, *p*-IκBα, TNF-α, Nrf2, and HO-1 proteins were normalized to those of β-actin (*n* = 3 per group). (C–G) (C) immunostaining of colonic tissues revealed various molecules indicated as green and then counterstained with DAPI indicated as blue: p-p65, TNF-α (scale bar, 200 μm). All representative images were captured using an inverted fluorescence microscope, levels of (D) SOD, (E) GSH-Px, (F) MPO, and (G) MDA in colonic tissues (*n* = 5 per group). Asterisk indicates statistically significant differences between control and experience group (∗∗p < 0.01; ∗∗∗p < 0.005).

To elucidate the potential mechanisms underlying the observed therapeutic efficacy, we further assessed the expression of key signaling pathway proteins. The NF-κB pathway is a key pro-inflammatory signal that triggers an inflammatory response in the progression of UC.^31^ The Nrf2 pathway regulates various detoxification enzymes and antioxidant proteins, such as HO-1, superoxide dismutase (SOD), and glutathione peroxidase (GSH-Px).^32^ In DSS-induced mice, levels of p-p65, *p*-IκBα, and TNF-α were significantly higher than those in the control group (Figures 5A and 5B). Treatment with IONPs, ZIF-8@IONPs, or HA@ZIF-8@IONPs effectively inhibited NF-κB pathway activation. Mice with DSS-induced colitis exhibited notably decreased expression levels of Nrf2 and HO-1 (Figures 5A and 5B) compared to normal control mice. Treatment with IONPs, ZIF-8@IONPs, or HA@ZIF-8@IONPs significantly restored the expression of these proteins (Figures 5A and 5B). Immunofluorescence analysis (Figure 5C) further supported the findings from the western blot assays.

SOD, myeloperoxidase (MPO), malondialdehyde (MDA), and GSH-Px are key indicators of oxidative stress in acute colitis.^33^ Figures 5D and 5E shows that DSS treatment significantly decreased SOD and GSH-Px activities in the colon relative to the control group. In contrast, DSS treatment significantly increased MDA and MPO activities (Figures 5F and 5G). Remarkably, administration of IONPs, ZIF-8@IONPs, or HA@ZIF-8@IONPs effectively reversed the DSS-induced alterations. Collectively, the smaller particle size and targeted delivery of HA@ZIF-8@IONPs contributed to enhanced therapeutic efficacy. The results support the hypothesis that a nanoplatform targeting the inflammatory microenvironment, coupled with antioxidative nano-iron oxide, provides a promising therapeutic approach for UC management.

### Conclusions

In summary, we demonstrated the ZIF-8 nanocarrier construct ZIF-8@IONPs for the treatment of UC, with additional HA encapsulation to enhance drug targeting. HA@ZIF-8@IONPs, this drug cleans ROS and relieves immune stress by its active component, IONPs. With the use of ZIF-8 carrier, the short action time and easy dispersion of IONPs alone can be remedied. The introduction of the HA, more make the whole drugs have targeted ability for inflammation. *In vivo* experiments showed that HA@ZIF-8@IONPs could reduce the damage of intestinal tissue and liver in DSS-induced UC model mice. In UC-induced mice, they demonstrated antioxidant and anti-inflammatory activity by eliminating endogenous ROS, activating Nrf2 signaling pathways, and inhibiting the NF-κB signaling pathway. To sum up, the HA@ZIF-8@IONPs with inflammatory bowel disease showed excellent curative effect can treat UC in mice.

### Limitations of the study

This study was conducted using DSS in female C57BL/6 mice, a well-established preclinical model of UC. However, the pathological manifestations of UC can vary across different animal models and between preclinical studies and human disease. Variations in genetic background, immune responses, and environmental factors may influence disease progression and treatment outcomes. Therefore, further investigations are needed to determine the applicability and translational relevance of our findings in other experimental models and human UC patients.

## Resource availability

### Lead contact

Further information and requests for resources and reagents should be directed to and will be fulfilled by the lead contact, Rong Wang (rongwang@nnu.edu.cn).

### Materials availability

All unique/stable reagents generated in this study are available from the lead contact with a completed materials transfer agreement.

### Data and code availability

• All datasets reported in this work are available from the lead contact upon request.
• No code was used in this work.
• Any additional information required to reanalyze the data reported in this paper is available from the lead contact upon request.

## Acknowledgments

This work was supported by National Natural Science Youth Foundation of China (82400609, 82302759).

## Author contributions

H.C.: conceptualization, data curation, methodology, writing an original draft. W.S. and C.L.: methodology, formal analysis, data curation. Q.W., X.W., and Y.D.: methodology, validation. W.C., M.W., C.H., and R.W.: conceptualization, funding acquisition, project administration, writing review and editing, supervision funding. All authors read and approved the final manuscript.

## Declaration of interests

The authors declare no competing interests.

## STAR★Methods

### Key resources table

**Table:** 

REAGENT or RESOURCE	SOURCE	IDENTIFIER
**Antibodies**		

Nrf2	Cell Signaling Technology	Cat#12721; RRID:AB_2715528
HO-1	Cell Signaling Technology	Cat# 86806; RRID:AB_2893444
TNF-α	Cell Signaling Technology	Cat# 11948; RRID:AB_2687962
p-p65	Cell Signaling Technology	Cat# 3033; RRID:AB_331284
*p*-IκBα	Cell Signaling Technology	Cat# 2859; RRID：AB_561111
Secondary antibody for IF	Abcam	Cat# ab150077; RRID:AB_2630356
Secondary antibody for WB	Abcam	Cat# ab205718; RRID:AB_2819160
β-Actin	Abcam	Cat# ab8226; RRID:AB_306371

**Biological samples**		

RAW 264.7 cells	Institute of Life Science Cell Culture Center in Shanghai	SCSP-5036
C57BL/6 mice	The Model Animal Research Center of Nanjing University	C57BL/6JGpt|strain NO.N00001
RAW 264.7 cells	Institute of Life Science Cell Culture Center in Shanghai	SCSP-5036
C57BL/6 mice	The Model Animal Research Center of Nanjing University	C57BL/6JGpt|strain NO.N00001

**Chemicals, peptides, and recombinant proteins**		

FeCl_3_·6H_2_O	Sigma Aldrich Life Sciences and High Technology Group	Cat#10025-77-1
FeCl_2_·4H_2_O	Sigma Aldrich Life Sciences and High Technology Group	Cat#13478-10-9
2-methylimidazole	Shanghai Aladdin Biochemical Technology Co., Ltd.	Cat#693-98-1
Zn(NO_3_)_2_·6H_2_O	Sinopharm Chemical Reagent Co., Ltd	Cat#10196-18-6
FeCl_3_·6H_2_O	Sigma Aldrich Life Sciences and High Technology Group	Cat#10025-77-1
FeCl_2_·4H_2_O	Sigma Aldrich Life Sciences and High Technology Group	Cat#13478-10-9
2-methylimidazole	Shanghai Aladdin Biochemical Technology Co., Ltd.	Cat#693-98-1
Zn(NO_3_)_2_·6H_2_O	Sinopharm Chemical Reagent Co., Ltd	Cat#10196-18-6

**Experimental models: Cell lines**		

RAW 264.7 cells	Institute of Life Science Cell Culture Center in Shanghai	SCSP-5036
RAW 264.7 cells	Institute of Life Science Cell Culture Center in Shanghai	SCSP-5036

### Experimental model and subject details

Female C57BL/6 mice, aged 6–8 weeks and weighing approximately 20 g, were obtained from the Model Animal Research Center of Nanjing University. The experimental protocols were approved by the Drum Tower Hospital committee, affiliated with the Medical School of Nanjing University, and were conducted in accordance with the Institutional Animal Care and Use Committee (IACUC) guidelines (Approval document: 2023AE02011). The mice were housed under specific pathogen-free (SPF) conditions at 22 ± 1°C with 50 ± 1% relative humidity and a 12-h light/dark cycle. UC was induced using oral dextran sulfate sodium (DSS).

### Method details

#### Materials

Polyglucose-sorbitol-carboxymethyl ether (PSC), dextran, FeCl_3_·6H_2_O, and FeCl_2_·4H_2_O were purchased from Sigma Aldrich Life Sciences and High Technology Group, Inc. 2-MA was obtained from Shanghai Aladdin Biochemical Technology Co., Ltd., and Zn(NO_3_)_2_·6H_2_O was sourced from Sinopharm Chemical Reagent Co., Ltd. All other chemicals were of analytical grade and were used as received unless specified otherwise.

#### Preparation of IONPs

PSC-coated IONPs were synthesized via chemical coprecipitation using ferrous chloride hexahydrate and tetrahydrate as precursors, following a previously described method.^15^ Briefly, 0.2 g of PSC was dissolved in 10 mL of deionized water. To this solution, 0.06 g of FeCl_3_ and 0.03 g of FeCl_2_, dissolved in 15 mL of deionized water, were added. The mixture was cooled to 5°C, after which 1 g of 28% (w/v) ammonium hydroxide was added, and the mixture was stirred for 2 min. The solution was then heated to 80°C for 1 h, followed by 24 h of dialysis with five ultrafiltration cycles using a 100 kDa membrane. The synthesized IONPs were stored at 4°C until further use.

#### Preparation of ZIF-8@IONPs

IONPs were incorporated into zeolitic imidazolate frameworks-8 (ZIF-8) to form a smart nanoplatform, designated as ZIF-8@IONPs, following a previously reported method. Briefly, 160 mg of Zn(NO_3_)_2_·6H_2_O was dissolved in 2 mL of water. Next, 0.261 mL of IONPs at a concentration of 23 mg/mL was prepared, and 2 g of 2-methylimidazole (2-MA) was dissolved in 10 mL of water. Both solutions were slowly combined and stirred at low speed for 12 h. After the reaction, the precipitate was collected by centrifugation, washed 1–2 times with water, and freeze-dried for 24 h to yield ZIF-8@IONPs in powder form.

#### Preparation of HA@ZIF-8@IONPs

First, 100 mg of hyaluronic acid (HA) and ZIF-8@IONPs were weighed and dissolved in 5 mL of water. The resulting solution was stirred vigorously for 48 h, then freeze-dried for an additional 24 h to obtain flocculent HA@ZIF-8@IONPs. The freeze-dried HA@ZIF-8@IONPs were stored at 4°C until further use.

#### Characterization

The as-prepared ZIF-8@IONPs and HA@ZIF-8@IONPs were primarily characterized using transmission electron microscopy (TEM) (JEM-200CX, JEOL, Japan). Hydrodynamic diameters and zeta potential were measured using dynamic light scattering (DLS) with a BeNano 90 Zeta particle size analyzer (China). X-ray diffraction (XRD) analysis was conducted using an Ultima IV diffractometer (Rigaku, Japan) to assess crystallinity. Fourier-transform infrared (FT-IR) spectra were recorded in attenuated total reflection (ATR) mode using a Thermo Fisher Nicolet iS 5 spectrometer, covering the range from 4000 to 500 cm^−1^ over 32 scans. Elemental distribution was analyzed using energy-dispersive spectroscopy (EDS) mapping.

#### *In vitro* study

##### Cell culture

RAW 264.7 cells were obtained from the Institute of Life Science Cell Culture Center in Shanghai, China. Cells were maintained in high-glucose Dulbecco’s Modified Eagle’s Medium (DMEM) supplemented with 10% fetal bovine serum and 1% penicillin/streptomycin at 37°C in a 5% CO_2_ incubator. To prevent spontaneous differentiation, cells were passaged frequently, and cell density was monitored to maintain growth within the range of 50%–80%. Subsequent experiments were performed using cells in the exponential growth phase.

##### Biocompatibility analysis

The biocompatibility of ZIF-8 and ZIF-8@IONPs with RAW 264.7 cells were assessed using the Cell Counting Kit-8 (CCK-8). RAW 264.7 cells were exposed to ZIF-8 or ZIF-8@IONPs at concentrations ranging from 0 to 50 μg/mL. After a 24-h incubation, 10% CCK-8 solution was added to each well, followed by an additional incubation for 30 min. Absorbance at 450 nm (A_450_) was then measured using a microplate reader.

##### Enzyme-linked immunosorbent assay (ELISA)

After homogenization and centrifugation (10000 ×g, 10 min, 4°C), the supernatant was collected and cryopreserved at −80°C. ELISA kits were adopted to assay the levels of inflammatory indicators (TNF-α). All ELISA procedures were implemented according to the kit protocols.

##### Evaluation of ROS

The ROS scavenging capacity of ZIF-8@IONPs was evaluated through fluorescence imaging. RAW 264.7 cells were exposed to 100 μM H_2_O_2_ for 90 min to induce oxidative stress and then incubated with ZIF-8@IONPs-0.5, ZIF-8@IONPs-1.0, or ZIF-8@IONPs-1.5 for 24 h. After media removal, each well was treated with 500 μL of a 10 μM DCFH-DA solution (Beyotime) and incubated for 30 min. The wells were then washed gently three times, and intracellular ROS levels were assessed by visualizing DCF green fluorescence signals using a fluorescence microscope.

##### Western Blot (WB) analysis

Colon samples were collected and immediately homogenized at 4°C. Protein concentrations were quantitatively measured using a BCA protein assay kit (Pierce Biotechnology, Rockford, IL, USA). Superoxide dismutase (SOD), myeloperoxidase (MPO), malondialdehyde (MDA), and glutathione peroxidase (GSH-Px) levels were assessed using assay kits from Nanjing Jiancheng Bioengineering Institute, following the manufacturer’s guidelines. Colonic tissues and cells were subjected to protein extraction using RIPA buffer (Beyotime Biotechnology, China), supplemented with a protease and phosphatase inhibitor cocktail (Invitrogen). SDS-polyacrylamide gel electrophoresis was performed according to established protocols. After electrophoresis, proteins were transferred to membranes, blocked with skim milk for 1 h, and incubated overnight with primary antibodies at 4°C. The membranes were then incubated with secondary antibodies for 1 h. Primary antibodies targeting Nrf2, HO-1, TNF-α, phospho-NF-κB p65, and phospho-IκBα were incubated at 4°C overnight. Chemiluminescence signals were visualized using an HRP substrate (Millipore, Germany) and detected with a Tanon 5200-Multi Chemiluminescent Imaging System (Tanon, China). β-actin served as a normalization control. Band density was semi-quantitatively analyzed using Image-Pro Plus software and presented in bar charts.

#### *In vivo* study

Female C57BL/6 mice, aged 6–8 weeks and weighing approximately 20 g, were obtained from the Model Animal Research Center of Nanjing University. The experimental protocols were approved by the Drum Tower Hospital committee, affiliated with the Medical School of Nanjing University, and were conducted in accordance with the Institutional Animal Care and Use Committee (IACUC) guidelines (Approval document: 2023AE02011). The mice were housed under specific pathogen-free (SPF) conditions at 22 ± 1°C with 50 ± 1% relative humidity and a 12-h light/dark cycle. UC was induced using oral dextran sulfate sodium (DSS). Mice were randomly assigned to five groups (*n* = 5 per group): normal control (sterile water), UC model (DSS), IONPs therapy (DSS + IONPs), ZIF-8@IONPs-1.0 therapy (DSS + ZIF-8@IONPs-1.0), and HA@ZIF-8@IONPs-1.0 therapy (DSS + HA@ZIF-8@IONPs-1.0). Colitis was induced by providing 3% DSS in the drinking water for 8 days. From Days 1–11 of the DSS treatment period, a daily intragastric dose of 100 mg/kg (w/w) of IONPs, ZIF-8@IONPs-1.0, or HA@ZIF-8@IONPs-1.0 was administered. Throughout the experimental period, daily assessments were conducted to monitor diet consumption, body weight, rectal bleeding, and fecal consistency. These parameters were assessed and scored according to established criteria to determine the disease activity index (DAI) for UC, providing a comprehensive measure of disease severity.

##### *In vivo* biocompatibility analysis

For histological analysis, the heart, liver, spleen, lungs, and kidneys were fixed in 10% formalin, embedded in paraffin, sectioned to a thickness of 5 μm, and mounted on slides. The sections were stained with H&E and examined under light microscopy.

##### *In vivo* imaging

Mice were administered oral gavage of Rhodamine B-labeled ZIF-8@IONPs and HA@ZIF-8@IONPs at a dose of 100 mg/kg body weight for three consecutive days. At 4 and 24 h post-final administration, the mice were euthanized, and their intestines were excised for evaluation of fluorescence intensity.

##### Histological analysis and serum biochemical test

For histological analysis, 0.5 cm segments of the distal colon were fixed in 10% formalin, embedded in paraffin, sectioned to a thickness of 5 μm, and mounted on slides. The sections were stained with H&E and examined under light microscopy. Histological damage scores were assigned based on established protocols. Mouse blood was collected and analyzed for serum biochemistry using an automatic biochemical analyzer (Chemray 420).

##### Immunofluorescence staining

For immunofluorescence analysis, after deparaffinization and gradient hydration, the tissue sections underwent antigen retrieval and blocking of endogenous peroxidase activity. A blocking buffer containing 10% fetal bovine serum (FBS) was applied to the slides for 1 h to prevent non-specific staining. The slides were then incubated overnight at 4°C with primary antibodies. The following day, the slides were incubated with fluorescence-conjugated secondary antibodies for 30 min and counterstained with DAPI for 5 min. The stained sections were visualized using an Olympus BX53 fluorescence microscope.

### Quantification and statistical analysis

All statistical analyses for dose-response data were performed using Origin 2017 or Graphpad Prism 8. For comparisons between experimental conditions, statistical significance was determined using two-way ANOVA or Student’s t test, where appropriate. All experiments were performed in duplicates or higher. The exact number of replicates (n) for each experiment is noted in the respective figure legends. All data are expressed as mean ± standard deviation (SD), unless otherwise noted in the figure legends.
