# Supplementary material for: Inflammatory targeted nanoplatform incorporated with antioxidative nano iron oxide to attenuate ulcerative colitis progression
Source: iScience. 2025 Apr 16;28(5):112448. doi: 10.1016/j.isci.2025.112448 (PMC12059676; doi:10.1016/j.isci.2025.112448)
Supplement: Document S1. Figures S1–S7 [file mmc1.pdf]

iScience, Volume *28*

Supplemental information

Inflammatory targeted nanoplatform incorporated with antioxidative nano iron oxide to attenuate ulcerative colitis progression

Haojun Chen, Wei Sun, Can Li, Qiuyang Wang, Xucai Wang, Yingjie Du, Wenbo Chen, Min Wang, Caoxing Huang, and Rong Wang

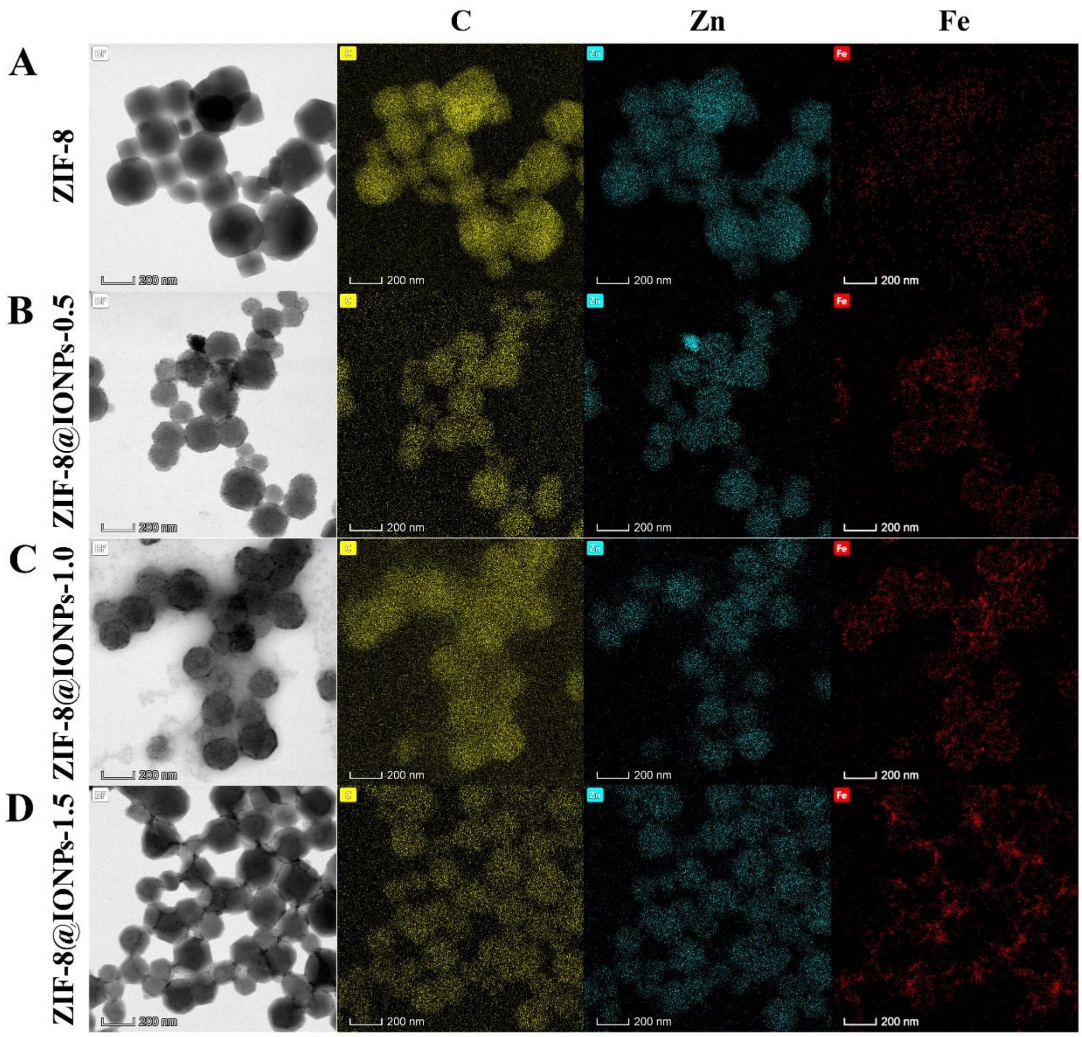

Fig. S1 Morphological characterization of various nanoplatform in detail. TEM images and their relevant element mapping of A.) ZIF-8. B.) ZIF-8@IONPs-0.5. C.) ZIF-8@IONPs-1. D.) ZIF-8@IONPs-1.5, respectively.

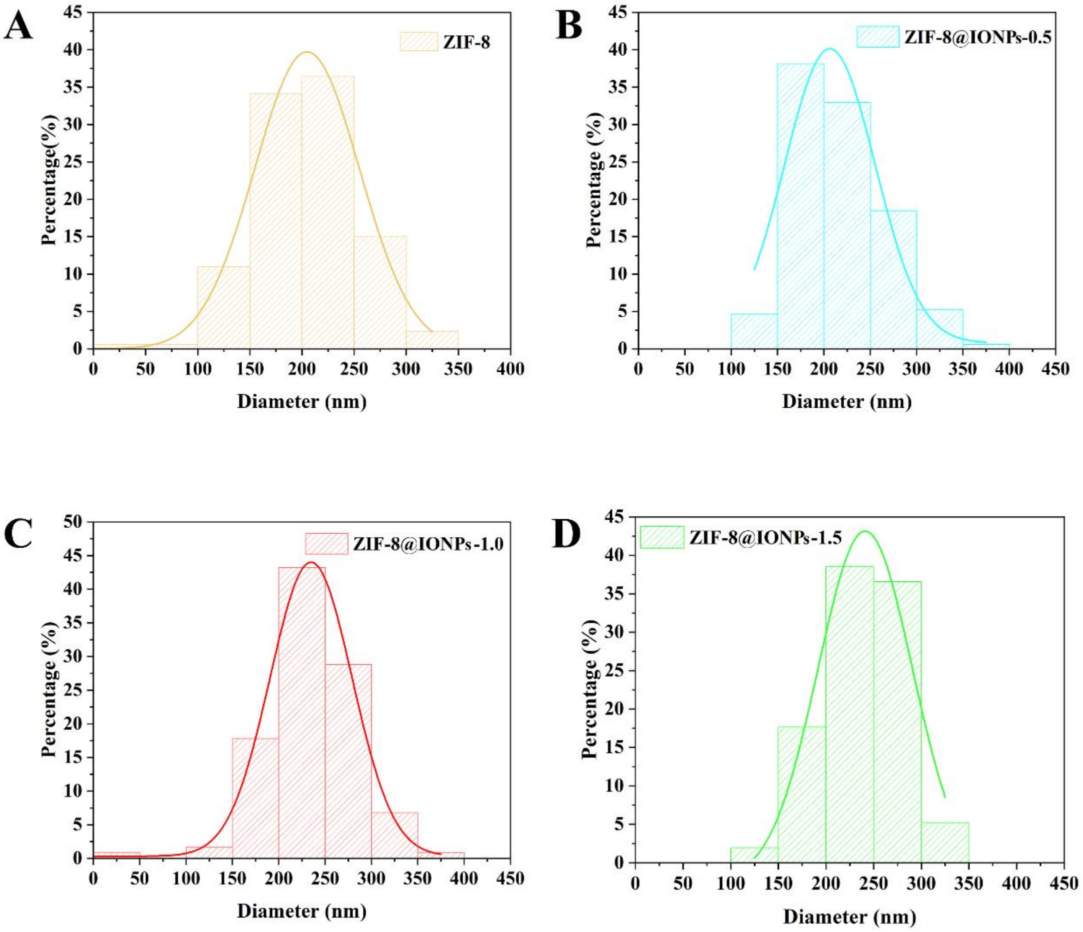

Fig. S2 Size distribution of various nanoplatform in detail. Size distribution histograms obtained from TEM images and their log-normal size distributions of A.) ZIF-8. B.) ZIF-8@IONPs-0.5. C.) ZIF-8@IONPs-1. D.) ZIF-8@IONPs-1.5, respectively.

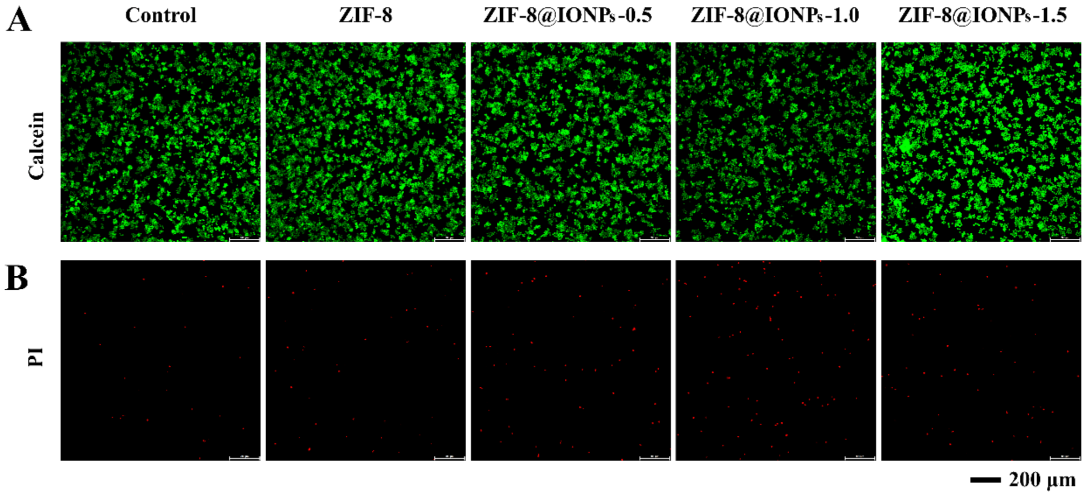

Fig. S3 Biocompatibility of various nanoplatform in detail. A.) Calcein-AM and B.) PI staining images of RAW 264.7 cells after co-incubated with ZIF-8, ZIF-8@IONPs-0.5, ZIF-8@IONPs-1 and ZIF-8@IONPs-1.5 for 24 hours, respectively.

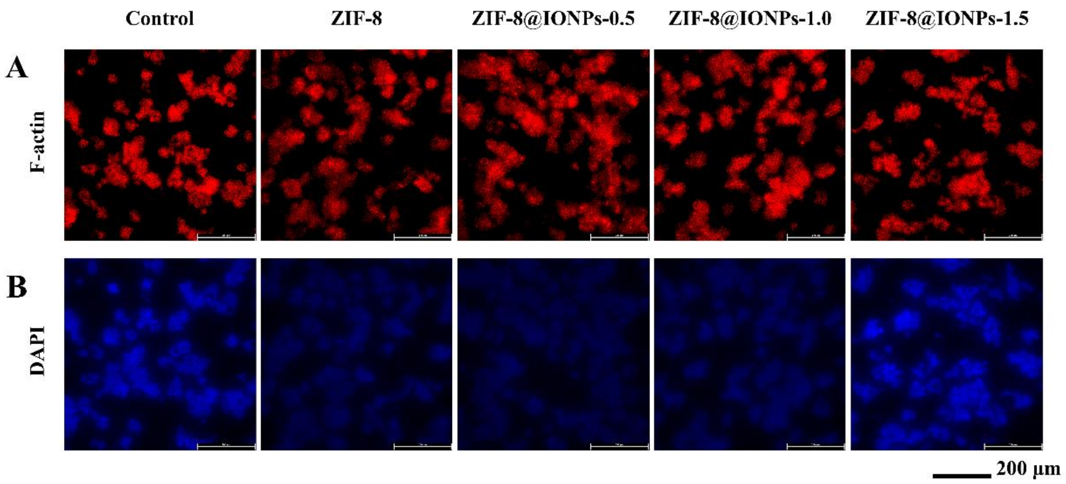

Fig. S4 Cell adhesion assessment of various nanoplatform in detail. A.) F-actin and B.) DAPI staining images of RAW 264.7 cells after co-incubated with ZIF-8, ZIF-8@IONPs-0.5, ZIF-8@IONPs-1 and ZIF-8@IONPs-1.5 for 24 hours, respectively.

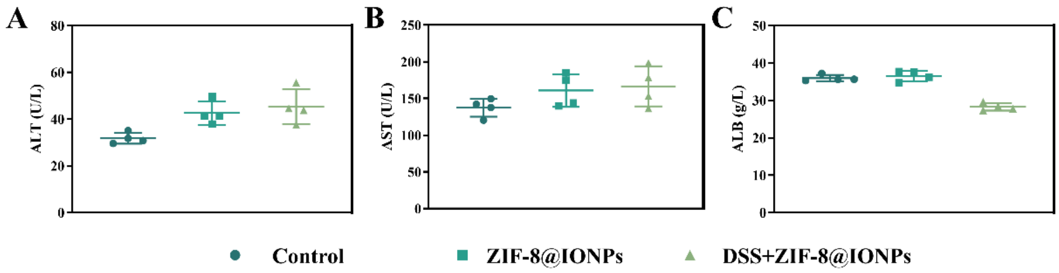

Fig. S5 Serum biochemical index assessment. A.) ALT, B.) AST and C.) ALB of mice intragastrically administered with control, ZIF-8@IONPs and DSS+ZIF-8@IONPs.

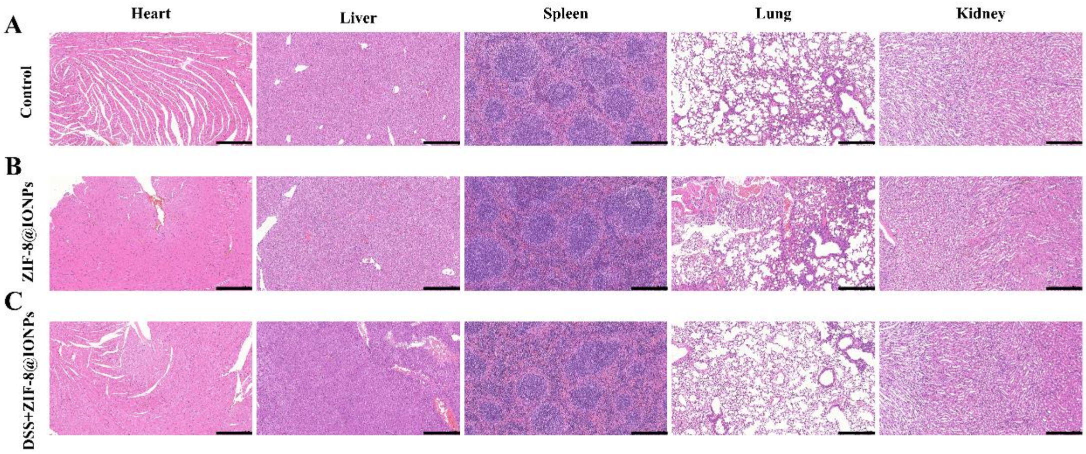

Fig. S6 Histological analysis. Representative H&E staining images of major organs in mice intragastrically administered with A.) control, B.) ZIF-8@IONPs and C.) DSS+ZIF-8@IONPs, respectively. (Scale bar = 400 μm).

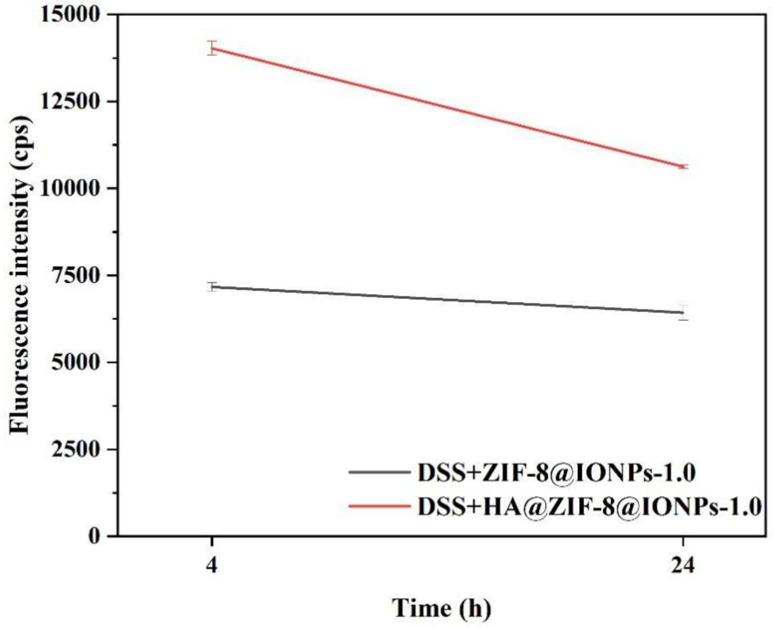

Fig. S7 Inflammatory targeting assessment. Quantification of the fluorescence distribution of rhodamine B-labeled ZIF-8@IONPs and HA@ZIF-8@IONPs in mice at different time points.
